# On the Relationship between Hydrogen Bond Strength and the Formation Energy in Resonance-Assisted Hydrogen Bonds

**DOI:** 10.3390/molecules26144196

**Published:** 2021-07-10

**Authors:** José Manuel Guevara-Vela, Miguel Gallegos, Mónica A. Valentín-Rodríguez, Aurora Costales, Tomás Rocha-Rinza, Ángel Martín Pendás

**Affiliations:** 1 Institute of Chemistry, National Autonomous University of Mexico, Circuito Exterior, Ciudad Universitaria, Delegación Coyoacán, Mexico City C.P. 04510, Mexico; jmguevarav@gmail.com (J.M.G.-V.); tomasrocharinza@gmail.com (T.R.-R.); 2 Department of Analytical and Physical Chemistry, University of Oviedo, 33006 Oviedo, Spain; gallegosmiguel@uniovi.es (M.G.); costalesmaria@uniovi.es (A.C.); 3 Instituto de Física Fundamental, Consejo Superior de Investigaciones Científicas (IFF-CSIC), Serrano 123, 28006 Madrid, Spain; monicavr@iff.csic.es

**Keywords:** hydrogen bond, interacting quantum atoms, resonance-assisted hydrogen bond

## Abstract

Resonance-assisted hydrogen bonds (RAHB) are intramolecular contacts that are characterised by being particularly energetic. This fact is often attributed to the delocalisation of π electrons in the system. In the present article, we assess this thesis via the examination of the effect of electron-withdrawing and electron-donating groups, namely −F, −Cl, −Br, −CF_3_, −N(CH_3_)_2_, −OCH_3_, −NHCOCH_3_ on the strength of the RAHB in malondialdehyde by using the Quantum Theory of Atoms in Molecules (QTAIM) and the Interacting Quantum Atoms (IQA) analyses. We show that the influence of the investigated substituents on the strength of the investigated RAHBs depends largely on its position within the π skeleton. We also examine the relationship between the formation energy of the RAHB and the hydrogen bond interaction energy as defined by the IQA method of wave function analysis. We demonstrate that these substituents can have different effects on the formation and interaction energies, casting doubts regarding the use of different parameters as indicators of the RAHB formation energies. Finally, we also demonstrate how the energy density can offer an estimation of the IQA interaction energy, and therefore of the HB strength, at a reduced computational cost for these important interactions. We expected that the results reported herein will provide a valuable understanding in the assessment of the energetics of RAHB and other intramolecular interactions.

## 1. Introduction

The structure and observable properties of condensed phases depend greatly on non-covalent interactions (NCI). The hydrogen bond (HB) is arguably the most important of these contacts, as it involved in many crucial phenomena in chemistry and biology, e.g., the association between DNA strands [1], enzymatic catalysis [2,3], molecular recognition [4,5], and protein folding [6]. HBs can result from the interaction of moieties in either different (intermolecular) or the same (intramolecular) molecules. Regarding the latter case, the formation of intramolecular HBs might produce profound changes in molecular properties and structure with far-reaching consequences. Indeed, intramolecular HBs can, for example, (i) alter membrane permeability, water solubility, and lipophilicity of molecules relevant in medicinal chemistry [7] or (ii) produce noticeable variations in the photochemical properties of molecules, such as shifts in the photoabsorption energy [8] or substantial variations in their photoisomerisation processes [9,10].

Resonance-assisted hydrogen bonds (RAHB) are very energetic HBs that are characterised by the connection between the proton donor and acceptor groups throughout conjugated double bonds. From their inception in the crystallographic work of Gill and coworkers [11,12,13,14,15], the concept of RAHB has been successfully adopted by the chemical community to explain phenomena in diverse fields such as physical [16,17,18] and organic chemistry [19,20,21] and in nuclear magnetic resonance [22,23].

Concerning the energetics of RAHBs, some authors consider the difference in energy between the associated closed and open conformers, also known as formation energy, as a measure of the strength of RAHBs [24]. Nevertheless, a problem that arises with this approach to study these interactions, and, as a matter of fact, with any other intramolecular contact, is that the rupture of the YH···X bond cannot occur without changing the structure of the molecule. Indeed, the above-mentioned method has the drawback that it involves the energy of (i) the HB itself and (ii) those corresponding to the changes taking place elsewhere in the molecule. In this context, different procedures to compute the interaction energy of RAHBs and other intramolecular non-covalent interactions have arisen [25,26]. Some of the most used methods are those that rely on the theoretical framework of the Quantum Theory of Atoms in Molecules (QTAIM) [27,28], such as the interacting quantum atoms (IQA) method of wave function analysis. The IQA approach involves an energy partition scheme that separates the total energy of an electronic system in intra- and interatomic terms [29,30]. Importantly, IQA allows for the univocal calculation of intramolecular interaction energies without requiring the definition of non-interacting fragments as opposed to traditional Energy Decomposition Analysis (EDA) methods [31]. IQA has been employed in the study of different intramolecular interactions in general [32,33,34,35,36] including, as a case of particular interest to this investigation, RAHBs [37,38,39,40].

In this article, we carried out electronic structure calculations as well as QTAIM and IQA analyses for a series of malondialdehydes substituted at different positions of the conjugated π system with an electron-withdrawing (EWG: −F, −Cl, −Br and −CF_3_) or an electron-donating (EDG: −N(CH_3_)_2_, −OCH_3_ and −NCOCH_3_) group [41]. These calculations enabled us (i) to study the effect of substituents in the strength of the RAHB depending on its relative position and (ii) to compare the values of the IQA interaction energies and other parameters from different EDA analyses on one hand, and the corresponding RAHB formation energies on the other. Our results show that in a considerable fraction of the examined cases, the formation energy deviates markedly from the IQA results. Therefore, such approaches to assess the strength of RAHBs are unable to properly differentiate contacts of this nature with very different energetic features. In contrast, the empirical formula proposed by Espinosa and coworkers [42,43] seems to adequately discern between RAHBs of different strengths. Overall, we expect the present investigation to yield novel insights about the different methods to compute the strength of RAHB and other relevant non-covalent intramolecular interactions.

## 2. Theoretical Framework

The QTAIM provides a division of space based on the topology of the electronic density. This method of wave function analysis enables the recovery of important chemical concepts, such as atoms, functional groups, atomic charges, and bond orders from either electronic structure calculations or X-ray experiments [44]. Consequently, QTAIM has been applied in the study of a wide variety of chemical and physical problems, such as the examination of different bonds [45,46], adsorption [47,48,49], electrical conductivity [50,51,52], and catalysis [53,54,55].

The traditional implementation of the IQA energy partition uses the atoms of QTAIM as a starting point to divide the total energy of an electronic system into the sum of self energies for each atom and interaction energies between the atoms in the system [29,30],

(1) $$\begin{array}{c} E=\sum_{A}E_{\mathrm{self}}^{A}+\sum_{A>B}E_{\mathrm{int}}^{AB}. \end{array}$$

$E_{\mathrm{self}}^{A}$ in Equation (1) is the energy corresponding to atom *A*, which includes its kinetic energy, the electron–nucleus attraction and the interelectronic repulsion within atom *A*. $E_{\mathrm{int}}^{AB}$ is the total interaction energy between atoms *A* and *B* and comprises all the possible combinations of the interaction terms between the nucleus and electrons of *A* on one hand, with the nucleus and electrons of *B* on the other.

We can also reorganise the terms included in $E_{\mathrm{int}}^{AB}$ in order to obtain an expression that gives us additional information about the nature of the interaction between *A* and *B*,

(2) $$\begin{array}{c} E_{\mathrm{int}}^{AB}=V_{\mathrm{cl}}^{AB}+V_{\mathrm{xc}}^{AB}. \end{array}$$

where $V_{\mathrm{cl}}^{AB}$ corresponds to the ionic part of the interaction energy while $V_{\mathrm{xc}}^{AB}$ is a term related with the covalency of the bond [56].

### 2.1. Models to Estimate the Energies of Intramolecular Hydrogen Bonds

The work dedicated to the estimation of the strength of intramolecular HBs has been very extensive, as reflected in the excellent review on the subject by Jabłoński [24]. Specifically, we will mainly focus on two indirect measurements. The first approach is based on the differences between the open and the closed conformations, referred to hereafter as the Open-Closed Method (OCM):

(3) $$\begin{array}{c} E_{\mathrm{HB}}^{\mathrm{intra}}\approx E_{\mathrm{form}}=E_{\mathrm{closed}}-E_{\mathrm{open}}. \end{array}$$

This methodology, albeit popular, presents two important drawbacks. First, it is not clear what geometry should be used as “open” [24]. For instance, Schuster has argued that the optimal open conformation for comparison purposes would be the one wherein minimal changes occur with respect to the closed conformation, even if its geometry is not a local minimum of the potential energy hypersurface [57]. We chose to use a different approach from that put forward by Schuster, and we considered optimised structures for both closed and open conformations of the systems under study. The other important drawback of the OCM method is that it combines changes taking place in other parts of the molecule with the energy corresponding to the HB itself [38]. Thus, stabilising and destabilising contributions, which result from other effects apart from the HB can be misattributed to this interaction. For example, steric destabilisation elsewhere in the molecule could be discounted from an examined intramolecular HB energy because it might occur that the HB is strong enough to compensate such unfavourable steric effects.

The second approximation, proposed by Espinosa et al. and denoted hereafter as Espinosa’s Method (EM), is based on the topology of the electronic density, specifically, on the correlation of the potential electron energy density at the bond critical point, $V(r_{\mathrm{bcp}})$, associated with a given HB and its corresponding energy according to the empirical expression [42,43],

(4) $$\begin{array}{c} E_{\mathrm{HB}}^{\mathrm{intra}}\approx E_{\mathrm{HB}}=\frac{1}{2}V\left(r_{\mathrm{bcp}}\right).\; \end{array}$$

This equation was put forward for the study of intermolecular HBs and has proven to be a suitable estimator for the formation energies as computed by the IQA approach in small and medium-sized water clusters, accounting for the relative order for the different types of HB contacts in these systems [58,59]. Nevertheless, some authors have questioned the uncritical use of EM for intramolecular HBs [60,61,62].

### 2.2. Computational Details

**Figure 1:**
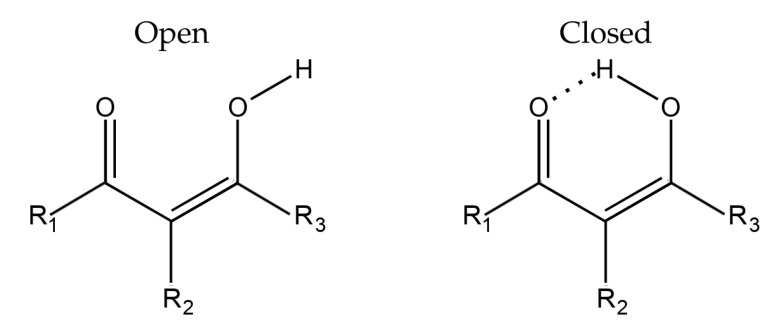
Malondialdehyde structure in its open (**left**) and closed (**right**) conformations. The R${}_{n}$ (n=1,2,3) symbols indicate the different positions available for substitution by the −F, −Cl, −Br, −CF_3_, −OCH_3_, −N(CH_3_)_2_, −NHCOCH_3_, and −NO_2_ groups.

We carried out electronic structure calculations for a series of derivatives of malondialdehyde in their open and closed configurations (Figure 1), where one of the hydrogens in the three carbon atoms of the conjugated skeleton is replaced by an EWG or an EDG, namely −F, −Cl, −Br, −CF_3_, −OCH_3_, −N(CH_3_)_2_, −NHCOCH_3_ or −NO_2_ (Figure 1). Thus, we computed the open and closed conformations for eight substitutions in three different positions resulting in 48 different structures wherein the RAHB is present in 24 of them. The conformers were chosen to minimise the differences between open and closed configurations and also to avoid secondary interactions, e.g., contacts between carbonyl and C-H groups. All the geometries were optimised with the aid of the B3LYP functional [63,64], along with the aug-cc-pVTZ basis set [65,66,67,68], as implemented in the Gaussian09 package [69]. This combination of exchange-correlation functional and basis set has yielded good results concerning the study of intramolecular hydrogen-bonded systems [40]. Harmonic frequency calculations were done in order to confirm that the optimised structures are indeed local minima. The QTAIM analyses were carried out with the help of the AimAll program [70]. The IQA energy partitions were carried out with our in-house Promolden code [71] using β-spheres with radii between 0.1 and 0.3 Bohr along with restricted angular Lebedev quadratures. We partitioned the exchange-correlation energy in accordance with Equation (1) via scaling techniques [72] previously used in conjunction with QTAIM.

## 3. Results

We present the main results of this investigation in three parts. First, the effect of the monosubstitution in malondialdehyde by the EWG and EDG considered in this work. Second, we will shed some light on the origin of the strong positional dependency of the substitution. Furthermore, third, we will compare the IQA results with the estimations put forward in Equations (3) and (4).

### 3.1. Influence of Substitution on RAHB Energetics

**Figure 2:**
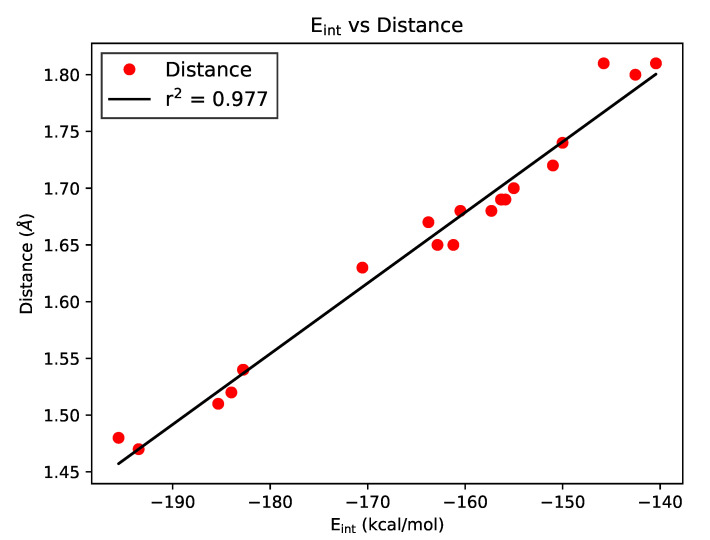
Correlation between IQA interaction energies in kcal/mol and OH···H distances in angstroms.

The IQA methodology partitions the energy of an electronic system into intra- and interatomic terms, a condition that allows the study of individual interactions within a molecule. An important characteristic of IQA is that this partition is carried out without using any reference system or empirical data. These features make IQA arguably the gold standard among the different methodologies, geometric or energetic alike, to study intramolecular interactions, including RAHBs. Figure 2 shows the excellent correlation between IQA interaction energies and the intramolecular hydrogen bond distance for the examined RAHBs.

The values for the interaction energy corresponding to the O···H contact with respect to those of malondialdehyde are reported in Table 1. The same chart reports the dissection of the IQA interaction energies into classical and exchange-correlation components. The relevance of the former over the latter contributions is conspicuous for malondialdehyde and the investigated EWGs and EDGs.

**Figure 3:**
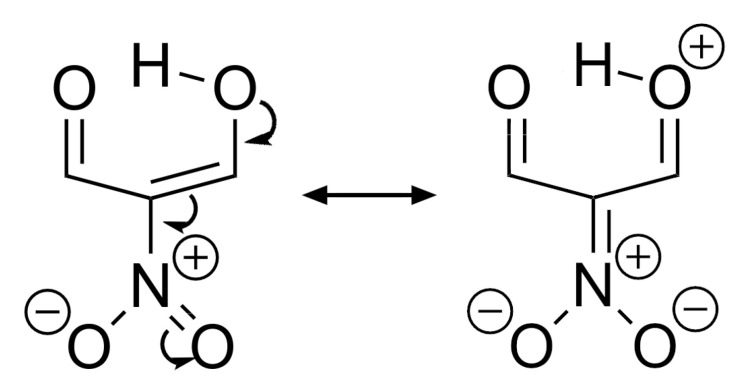
Resonance effect of an electron withdrawing substituent at position 2 in the examined RAHBs.

We note that the monosubstitution in different positions can either weaken or strengthen the associated RAHB. For example, the −CF_3_ group weakens the RAHB when it is bonded directly to the carbonyl group, but it has the opposite effect in positions 2 and 3. Concerning the halogens, they decrease the intensity of the O···H interaction when they are located in positions 1 and 2. On the other hand, they decrease the magnitude of the interaction energy when they are bonded to the enolic carbon. Mesomeric structures suggest that the influence of EWGs via resonance would be more noticeable on the RAHB strength when the EWG is bonded to the α carbon (Figure 3). The effect of the EWG are therefore more likely interpreted to occur via inductive effects. Furthermore, the influence of these groups is most obvious when they are close to the HB donor. Nevertheless, Table 1 shows that the exchange-correlation contribution to bonding also increases when EWG is bonded to the β carbon, and thus resonance effects cannot be completely neglected.

**Figure 4:**
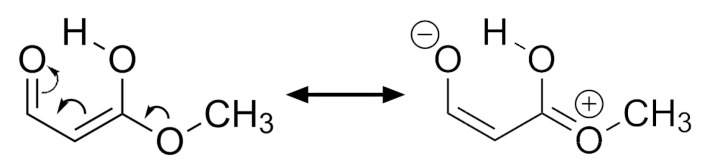
Resonance effect of an electron donating substituent at position 3 in the examined RAHBs.

With respect to the examined EDG, we note that these groups have a minimal effect (a slight reduction) when they are located at position 2. Notwithstanding when they are at position 1 and especially at position 3, they notably increase the RAHB interaction energy. This effect can be understood in terms of the mesomeric structures shown in Figure 4. Interestingly, the substitution in position 3 has the most conspicuous influence effect, leading in all cases to a strengthening of the interaction.

The above-mentioned effects can be used as guidelines by synthetic chemists to modulate the strength of RAHBs, via the electron withdrawing or donating features of a given substituent, together with its position in the conjugated system.

### 3.2. Comparison between IQA, OCM, and EM Methods

**Figure 5:**
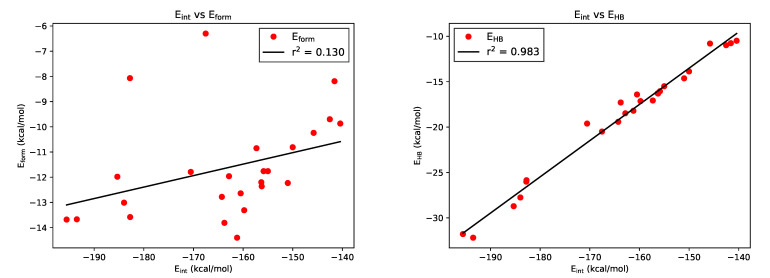
Correlation of IQA interaction energies with (**left**) OCM values of $E_{\mathrm{form}}$ and (**right**) EM results of $E_{\mathrm{HB}}$.

Table 2 reports the different assessments of the RAHB energy considered in this paper, namely, IQA, OCM, and EM. Figure 5 shows the relationship between (i) the IQA interaction energy and (ii) the formation energy computed with the OCM method together with the HB energy calculated using EM. As we can appreciate from the left side of Figure 5, the IQA interaction energy is not correlated with the $E_{\mathrm{form}}$ results yielded by the OCM approach. The fact that these results are unconnected can be associated with the main thesis of this work: the breaking of an RAHB can trigger a rearrangement in the electronic density, which is unrelated to the energetic features of the investigated RAHB [38]. Indeed, these unavoidable modifications take place in molecular regions unrelated to the RAHB. This fact makes the OCM ($E_{\mathrm{form}}$) unsuitable as a parameter for the assessment of the formation energies of the RAHBs under consideration.

Contrary to this fact, the correlation between the values of $E_{\mathrm{int}}$ and those corresponding to Equation (4) are excellent. In all cases, IQA interaction energies and Espinosa’s empirical formula produce indeed the same relative strengths for the studied systems. This observation indicates that for typical RAHBs, Equation (4) is able to qualitatively recover the interplay between the π-skeleton and the O···H−O moiety.

The good agreement between $E_{\mathrm{int}}$ and $E_{\mathrm{HB}}$ should not be interpreted as an uncritical approval to the use of Equation (4) for the estimation of relative RAHB strengths. Certainly, different authors have pointed out a series of deficiencies for this empirical formulation. Here we mention two of these potential problems. First, the values resulting from Equation (4) are always negative, a circumstance that always points to an attractive interaction. Nevertheless, certain C−H···O contacts are repulsive in nature [62]. The use of Formula (3) to describe these contacts would produce a qualitatively incorrect result. Second, Equation (4) is not transferable to other contacts, such as H···F, where a different scaling factor from that used in Equation (4) needs to be used [73].

We finally state what we consider the limits for the reasonable application of EM on the study of intramolecular HBs in general and RAHB in particular. Given that Equation (4) is unable to distinguish between attractive and repulsive interactions, it should be only applied when no doubt can arise regarding the attractive nature of the contact. Additionally, although $E_{\mathrm{int}}$ and $E_{HB}$ follow the same strength order, their magnitudes are not comparable. Therefore, the EM should primarily be used to study intramolecular contacts where the interaction mainly involve the same atomic species. This situation might be the case, for instance, in different substitutions in an aromatic ring adjacent to the O−H···O group [74] or changes in the protonation degree in an intramolecular HB [75].

## 4. Conclusions

We investigate the effect of different electron-withdrawing and -donor groups on the energetics of the resonance assisted hydrogen bond in malondialdehyde. Our data indicate that classical contributions are far more important than exchange-correlation components as opposed to the notion that the stability of RAHBs occur mainly due to the delocalisation of π electrons. These groups exert a marked influence on the RAHB interaction energy, which in turn depends notably on the position of the EWG and EDG. Notably, both types of groups considerably strengthen the RAHB when they are bonded to the β carbon atom of malondialdehyde. We also addressed different methodologies to assess the interaction energy of RAHBs. In this regard, we showed how the examination of the energy density offers a good estimation of the IQA interaction energy and therefore of the RAHB energetics at a reduced computational cost.

## Figures and Tables

**Table 1:** IQA interaction energies ($E_{\mathrm{int}}$), as well as its classical ($V_{\mathrm{cl}}$) and exchange-correlation ($V_{\mathrm{xc}}$) parts, for the investigated O···H RAHB contacts with respect to those in malondialdehyde (Table S1). The values are reported in kcal·mol${}^{-1}$.

	R${}_{1}$			R${}_{2}$			R${}_{3}$		
**–R**	**$E_{\mathrm{int}}$**	**$V_{\mathrm{cl}}$**	**$V_{\mathrm{xc}}$**	**$E_{\mathrm{int}}$**	**$V_{\mathrm{cl}}$**	**$V_{\mathrm{xc}}$**	**$E_{\mathrm{int}}$**	**$V_{\mathrm{cl}}$**	**$V_{\mathrm{xc}}$**
–CF_3_	8.78	6.22	2.57	−1.43	−0.90	−0.53	−3.06	−2.25	−0.80
–F	13.99	8.11	5.88	9.77	6.97	2.80	−33.71	−21.09	−12.62
–Cl	17.24	11.36	5.88	3.91	2.68	1.24	−24.20	−15.19	−9.02
–Br	19.35	12.93	6.41	3.53	2.45	1.08	−25.55	−15.70	−9.85
–N(CH_3_)_2_	−10.76	−9.02	−1.74	3.45	2.35	1.10	−35.77	−23.84	−11.92
–OCH_3_	−3.99	−4.22	0.23	4.77	3.33	1.44	−22.97	−15.63	−7.34
–NCOCH_3_	−0.71	−1.73	1.03	2.47	1.81	0.67	−23.01	−15.57	−7.43
–NO_2_	18.20	12.11	6.09	−4.50	−2.84	−1.66	−7.75	−5.09	−2.67

**Table 2:** IQA interaction energies ($E_{\mathrm{int}}$), formation energies ($E_{\mathrm{form}}$) computed by means of the OCM (expression (3)), and H-bond interaction energies ($E_{\mathrm{HB}}$) estimated via Equation (4) for the investigated O···H RAHB contacts. The values are reported in kcal·mol${}^{-1}$.

	R${}_{1}$			R${}_{2}$			R${}_{3}$		
**–R**	**$E_{\mathrm{int}}$**	**$E_{\mathrm{form}}$**	**$E_{\mathrm{HB}}$**	**$E_{\mathrm{int}}$**	**$E_{\mathrm{form}}$**	**$E_{\mathrm{HB}}$**	**$E_{\mathrm{int}}$**	**$E_{\mathrm{form}}$**	**$E_{\mathrm{HB}}$**
–CF_3_	8.78	1.08	2.51	−1.43	−1.09	−1.06	−3.06	1.35	−1.34
–F	13.99	3.07	6.34	9.77	2.50	3.27	−33.71	−0.36	−15.04
–Cl	17.24	3.61	6.16	3.91	1.55	1.09	−24.20	0.30	−10.61
–Br	19.35	3.44	6.65	3.53	0.95	0.84	−25.55	1.33	−11.57
–N(CH_3_)_2_	−10.76	1.52	−2.47	3.45	1.11	0.86	−35.77	−0.37	−14.64
–OCH_3_	−3.99	−0.50	−0.15	4.77	1.55	1.64	−22.97	−0.27	−8.71
–NCOCH_3_	−0.71	0.67	0.73	2.47	2.46	0.07	−23.01	5.24	−8.87
–NO_2_	18.20	5.12	6.38	−4.50	0.53	−2.28	−7.75	7.01	−3.35

## Data Availability

Not applicable.

## Supplementary Materials

The following are available online, Table S1: IQA interaction energies for the investigated systems; Table S2: IQA interaction, formation, and HB interaction energies. Figures S1–S8: Structures of all compounds.
