# Supplementary material for: On the Relationship between Hydrogen Bond Strength and the Formation Energy in Resonance-Assisted Hydrogen Bonds
Source: Molecules. 2021 Jul 10;26(14):4196. doi: 10.3390/molecules26144196 (PMC8303970; doi:10.3390/molecules26144196)
Supplement: Supplementary file 1 [file molecules-26-04196-s001.zip › supp.pdf]

On the relationship between hydrogen bond strength and the formation energy in resonance-assisted hydrogen bonds

Electronic Supplementary Information

J. M. Guevara-Vela, M. Gallegos, M. A. Valentín-Rodríguez,
A. Costales, T. Rocha-Rinza, Á. Martín Pendás*

June 30, 2021

*To whom correspondence should be addressed: ampendas@uniovi.es

Table S1: IQA interaction energies (E_{int}) as well as its classical (V_{cl}) and exchange-correlation (V_{xc}) parts, for the investigated O···H RAHB contacts. The values are reported in kcal·mol^{-1}.

	Position 1			Position 2			Position 3		
System	E_{int}	V_{cl}	V_{xc}	E_{int}	V_{cl}	V_{xc}	E_{int}	V_{cl}	V_{xc}
–CF_3	−151.00	−133.97	−17.02	−161.21	−141.09	−20.12	−162.84	−142.44	−20.39
–F	−145.79	−132.08	−13.71	−150.01	−133.22	−16.79	−193.49	−161.28	−32.21
–Cl	−142.54	−128.83	−13.71	−155.87	−137.51	−18.35	−183.98	−155.38	−28.61
–Br	−140.43	−127.26	−13.18	−156.25	−137.74	−18.51	−185.33	−155.89	−29.44
–$N(CH_3)_2$	−170.54	−149.21	−21.33	−156.33	−137.84	−18.49	−195.55	−164.03	−31.51
–OCH_3	−163.77	−144.41	−19.36	−155.01	−136.86	−18.15	−182.75	−155.82	−26.93
–$NCOCH_3$	−160.49	−141.92	−18.56	−157.31	−138.38	−18.92	−182.79	−155.76	−27.02
–NO_2	−141.59	−128.08	−13.51	−164.28	−143.03	−21.25	−167.54	−145.27	−22.26
Malondialdehyde	−159.78	−140.19	−19.59						

Table S2: IQA interaction energies (E_{int}), formation energies (E_{form}) computed by means of the OCM (expression (3)), and H-bond interaction energies (E_{HB}) estimated via equation (4) for the investigated O···H RAHB contacts. The values are reported in kcal·mol^{-1}.

	Position 1			Position 2			Position 3		
System	E_{int}	E_{form}	E_{HB}	E_{int}	E_{form}	E_{HB}	E_{int}	E_{form}	E_{HB}
–CF_3	−151.00	−12.23	−14.63	−161.21	−14.40	−18.20	−162.84	−11.96	−18.48
–F	−145.79	−10.24	−10.80	−150.01	−10.81	−13.87	−193.49	−13.67	−32.18
–Cl	−142.54	−9.70	−10.98	−155.87	−11.76	−16.05	−183.98	−13.01	−27.75
–Br	−140.43	−9.87	−10.49	−156.25	−12.36	−16.30	−185.33	−11.98	−28.71
–$N(CH_3)_2$	−170.54	−11.79	−19.61	−156.33	−12.20	−16.28	−195.55	−13.68	−31.78
–OCH_3	−163.77	−13.81	−17.29	−155.01	−11.76	−15.50	−182.75	−13.58	−25.85
–$NCOCH_3$	−160.49	−12.64	−16.41	−157.31	−10.85	−17.07	−182.79	−8.07	−26.01
–NO_2	−141.59	−8.19	−10.76	−164.28	−12.78	−19.42	−167.54	−6.30	−20.49
Malondialdehyde	−159.78	−13.31	−19.59						

1 Structures of the studied systems

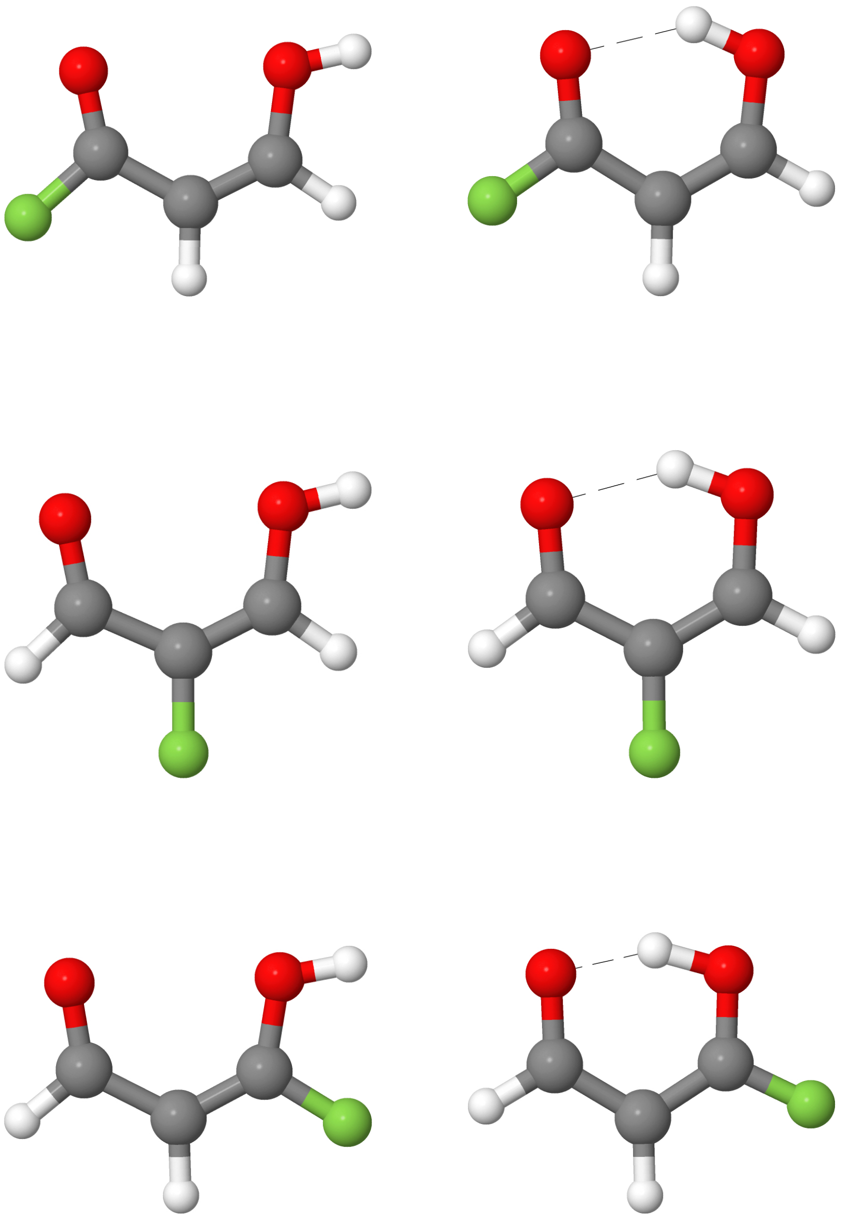

Figure S1: Structures of the open (left) and closed (right) configurations of malondialdehyde substituted by –F on the positions 1 (top), 2 (middle) or 3 (bottom).

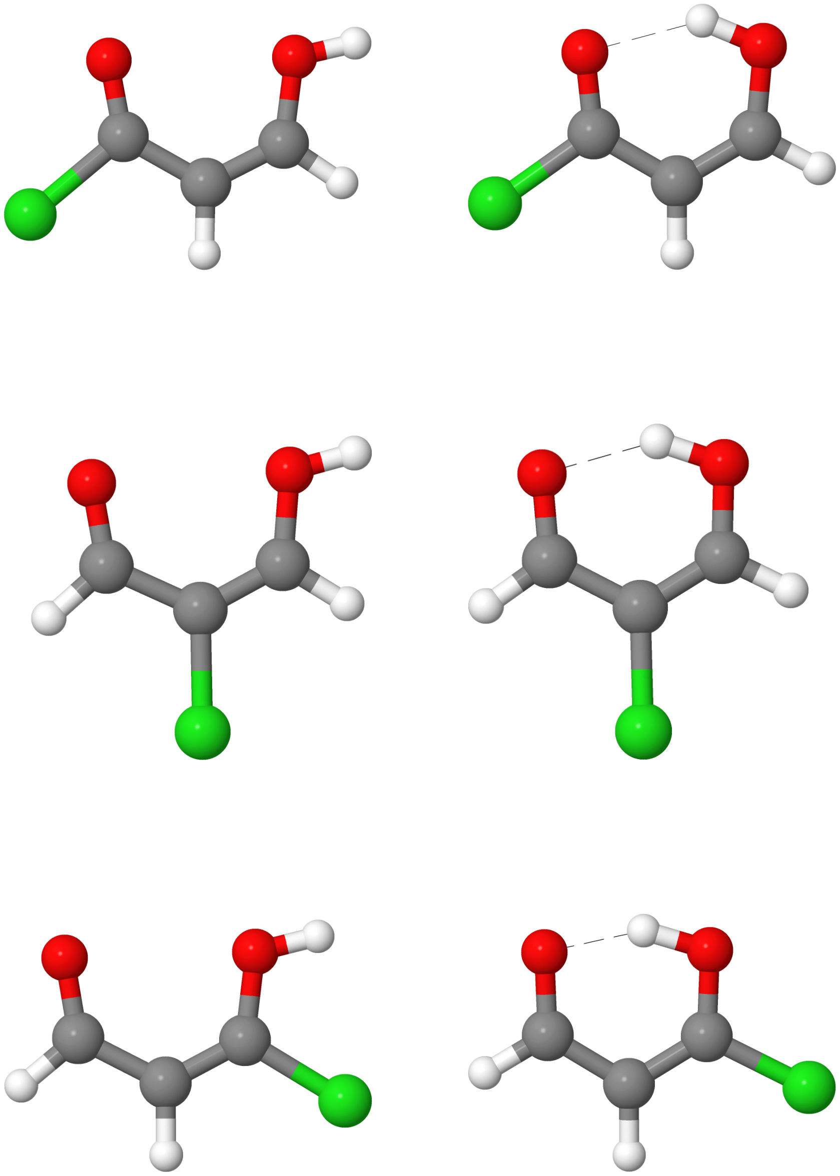

Figure S2: Structures of the open (left) and closed (right) configurations of malondialdehyde substituted by –Cl on the positions 1 (top), 2 (middle) or 3 (bottom).

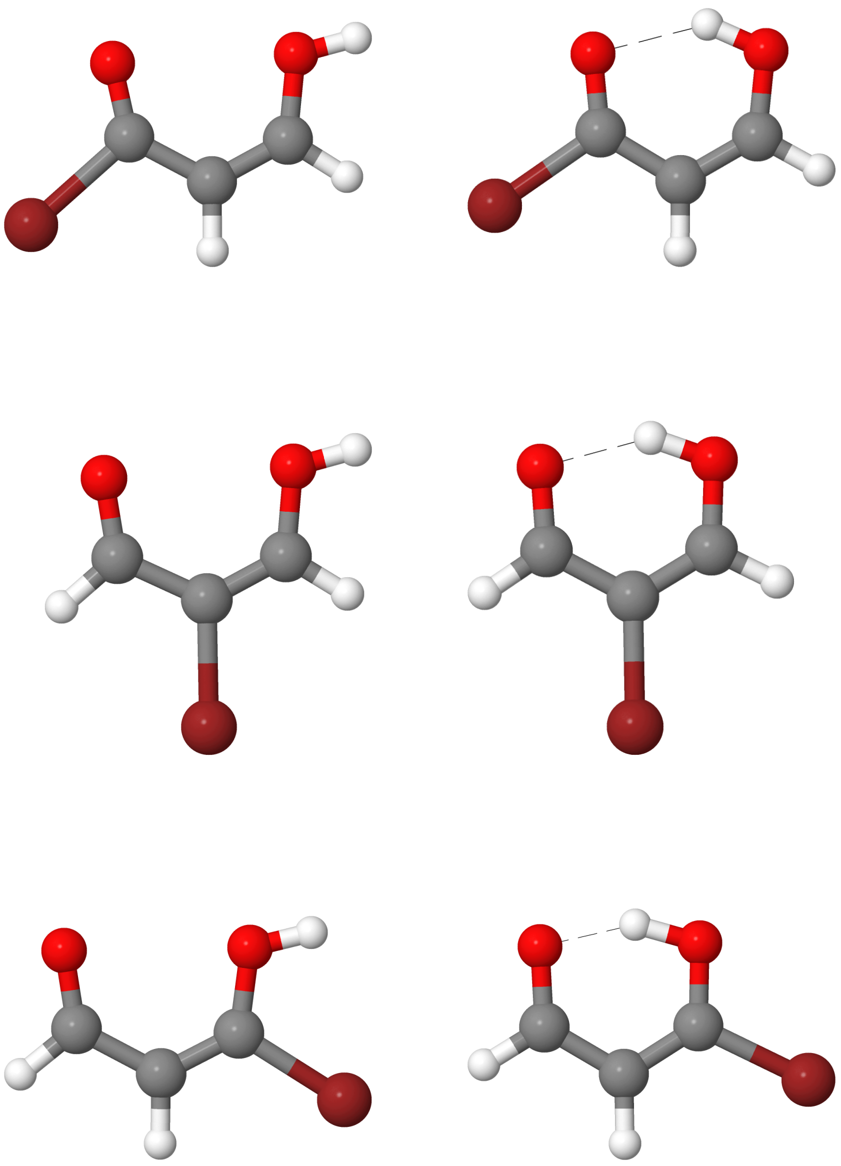

Figure S3: Structures of the open (left) and closed (right) configurations of malondialdehyde substituted by –Br on the positions 1 (top), 2 (middle) or 3 (bottom).

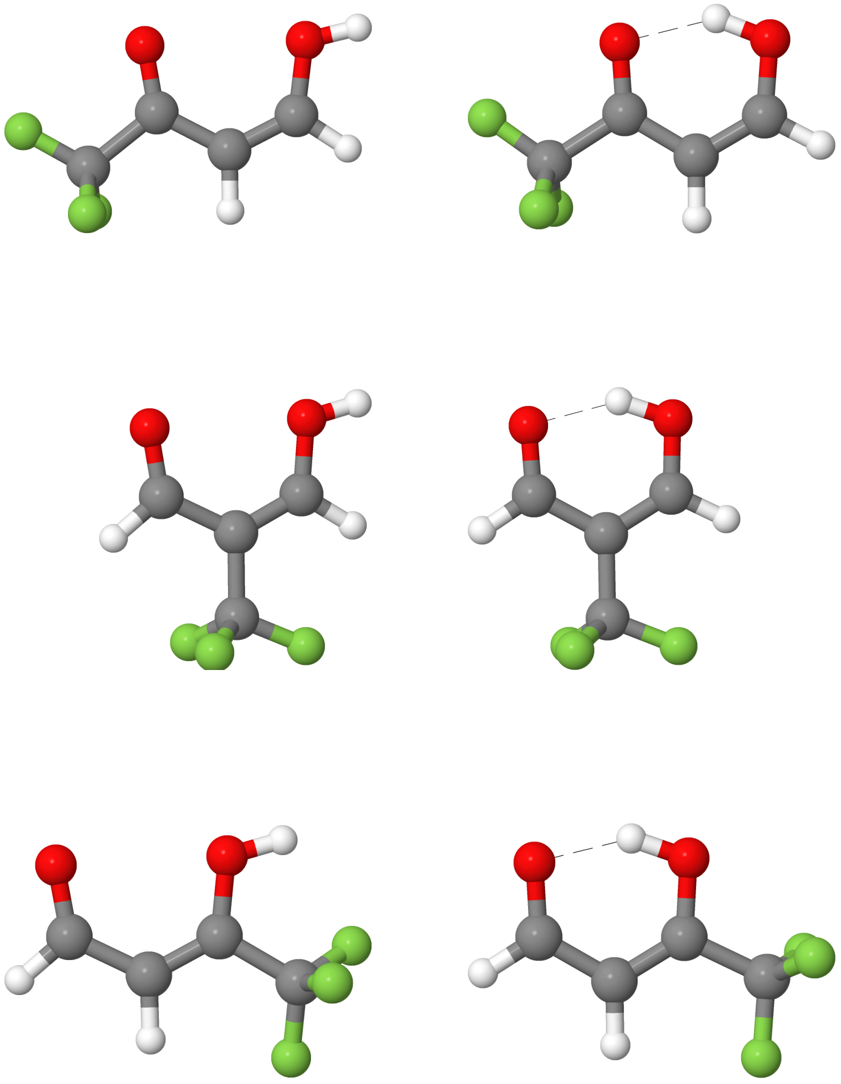

Figure S4: Structures of the open (left) and closed (right) configurations of malondialdehyde substituted by –CF_3 on the positions 1 (top), 2 (middle) or 3 (bottom).

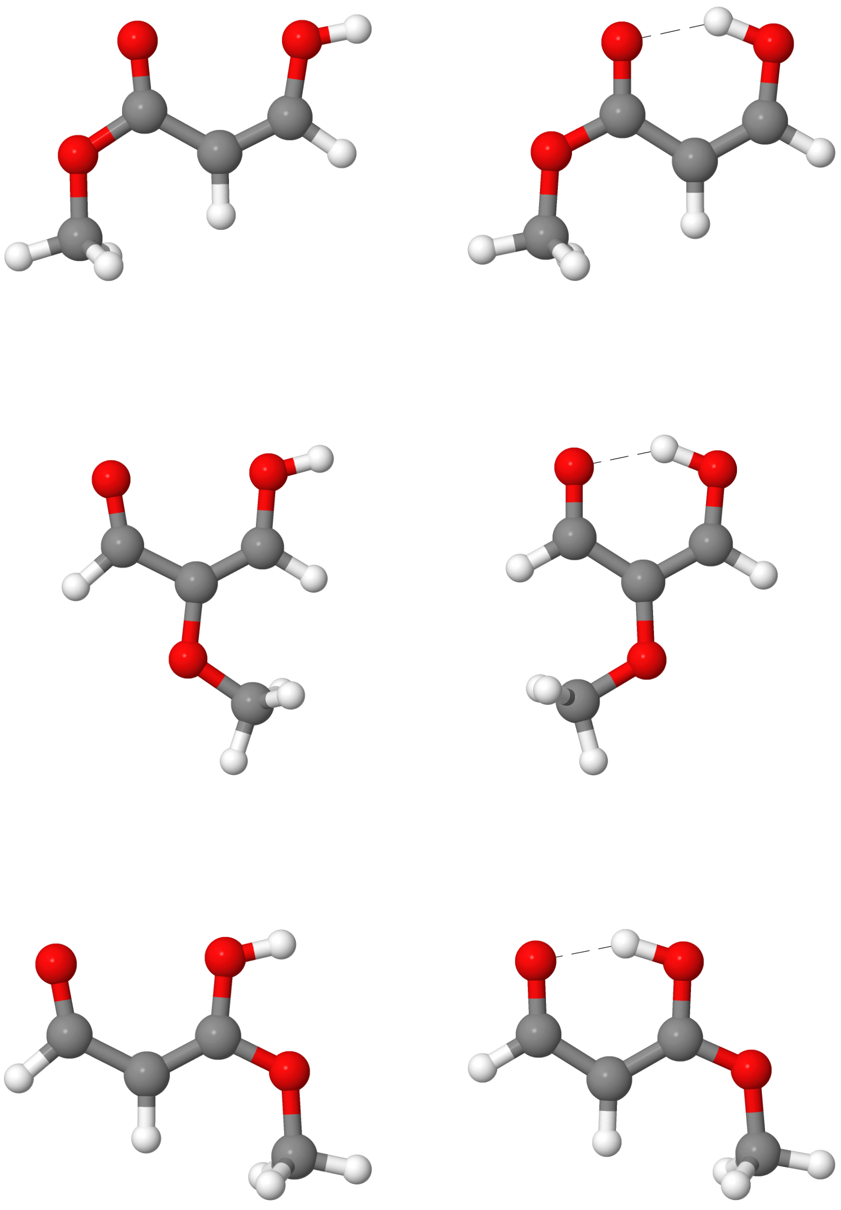

Figure S5: Structures of the open (left) and closed (right) configurations of malondialdehyde substituted by the metoxi group on the positions 1 (top), 2 (middle) or 3 (bottom).

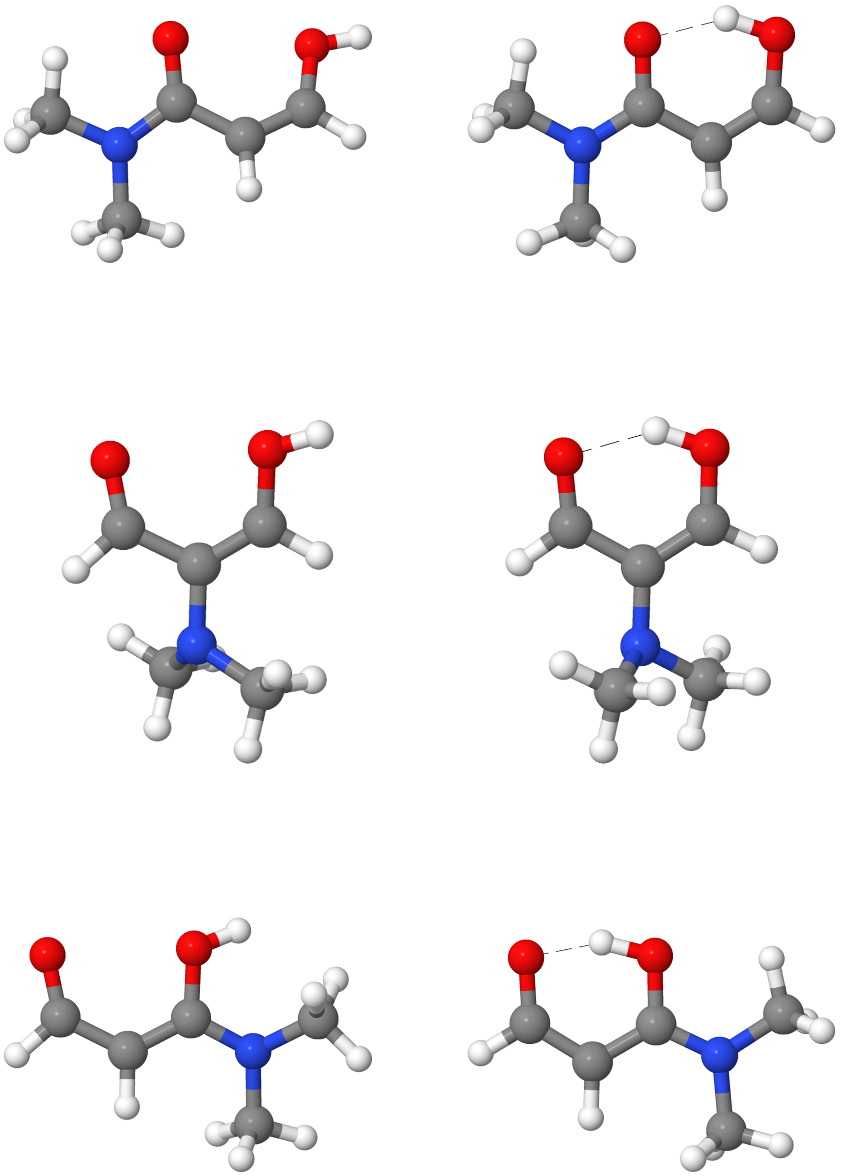

Figure S6: Structures of the open (left) and closed (right) configurations of malondialdehyde substituted by the dimetilamine group on the positions 1 (top), 2 (middle) or 3 (bottom).

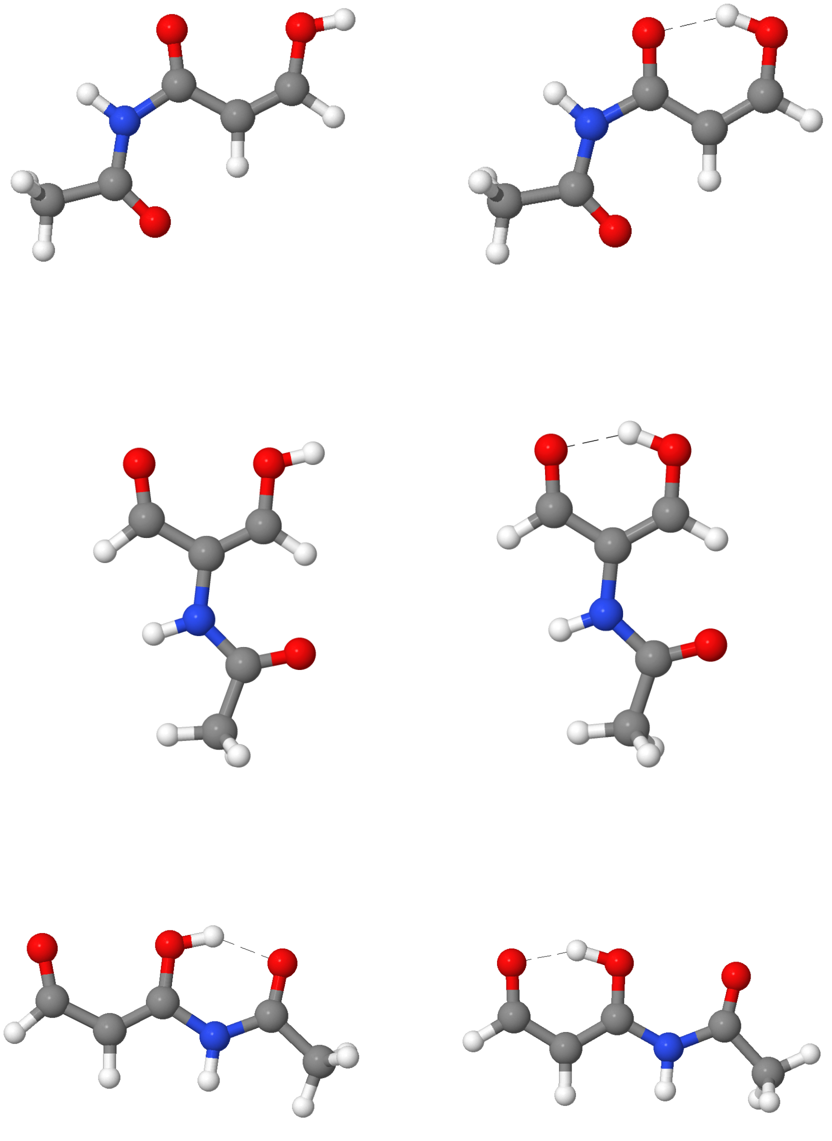

Figure S7: Structures of the open (left) and closed (right) configurations of malondialdehyde substituted by the ethanamide group on the positions 1 (top), 2 (middle) or 3 (bottom).

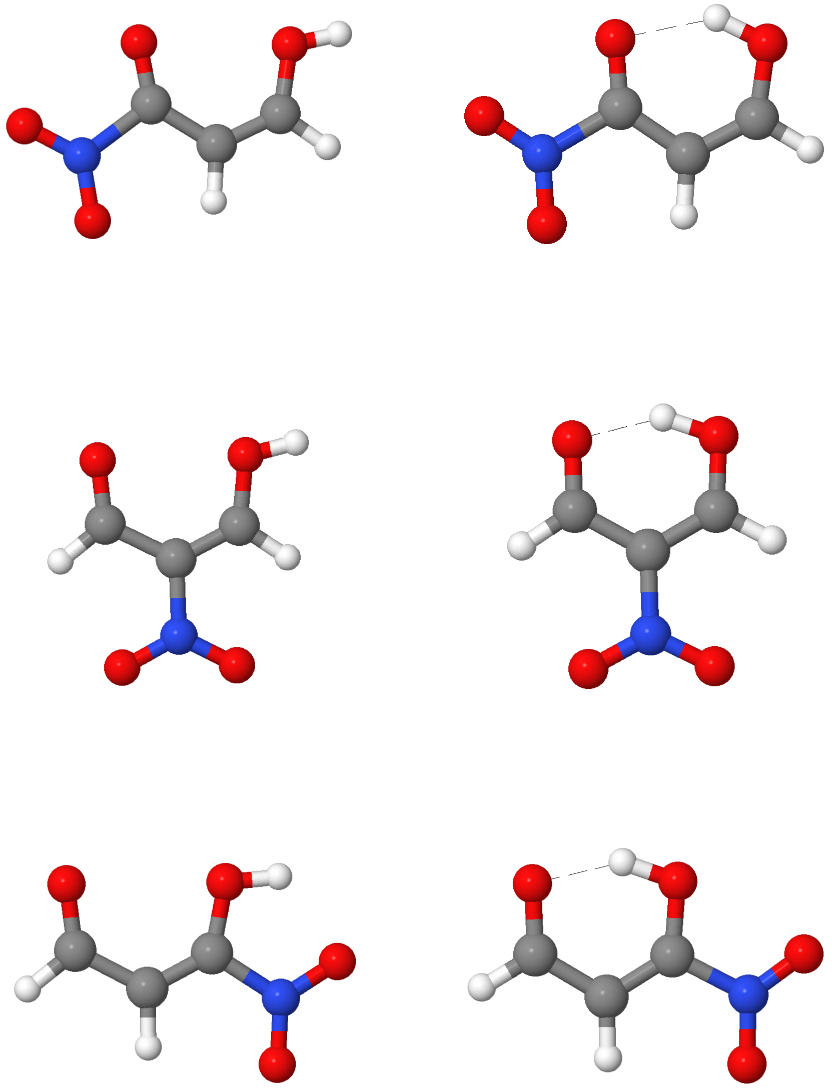

Figure S8: Structures of the open (left) and closed (right) configurations of malondialdehyde substituted by the nitro group on the positions 1 (top), 2 (middle) or 3 (bottom).
